# Human Health Risk Assessment through Roasted Meats Consumption

**DOI:** 10.3390/ijerph17186737

**Published:** 2020-09-16

**Authors:** Luana C. S. Leite, Elaine S. de P. Melo, Daniela G. Arakaki, Elisvânia F. dos Santos, Valter A. do Nascimento

**Affiliations:** 1 Group of Spectroscopy and Bioinformatics Applied to Biodiversity and Health, School of Medicine, Postgraduation Program in Health and Development in the Midwest Region, Faculty of Medicine, Federal University of Mato Grosso do Sul, Campo Grande MS 79079-900, Brazil; luanacarolinasl@gmail.com (L.C.S.L.); elainespmelo@hotmail.com (E.S.d.P.M.); daniarakaki@gmail.com (D.G.A.); 2 Faculty of Pharmaceutical Sciences, Food, and Nutrition, Federal University of Mato Grosso do Sul—UFMS, Campo Grande MS 79079-900, Brazil; elisvania@gmail.com; 3 Postgraduation Program in Health and Development in the Midwest Region, Faculty of Medicine, Federal University of Mato Grosso do Sul, Campo Grande MS 79079-900, Brazil

**Keywords:** toxic metals, chronic daily intake, human health risk assessment, ICP-OES, arsenic

## Abstract

Data on the content of metals and metalloids in roasted meats with different types of wood and charcoal are still scarce in the literature. The concentrations of metals (Al, Cr, Cd, Cu, Fe, Mg, Mn, Mo, Ni, V, and Zn) and metalloid (As) were determined by inductively coupled plasma mass spectrometry (ICP-OES) after microwave digestion, and the estimated daily intake (*EDI*) for adults was assessed to determine the hazard quotient (*HQ*). The concentrations of Al, Cr, Cu, and Fe in raw meats were below the data obtained in other countries. The concentration of As (0.17 ± 0.42–0.23 ± 0.10 mg/kg), Mg (206.77 ± 3.99–291.95 ± 8.87 mg/kg), V (0.42 ± 0.14–6.66 ± 0.80 mg/kg), and Zn (6.66 ± 0.80–48.13 ± 0.56 mg/kg) in raw meats exceeded the values in the literature. The concentrations of Mg, As, Cr, Fe, V, and Zn are high when the meat is roasted using wood. All levels of Al, As, Cr, Cu, Fe, Mg, Mn, Mo, V, and Zn in raw meats are lower than those of meat roasted with coal and wood. The content of As in meat roasted with Chromed Copper Arsenate (CCA) wood (15.10 ± 0.27–26.25 ± 1.47 mg/kg) is higher than meat roasted with charcoal (0.46 ± 0.09–1.16 ± 0.50 mg/kg). *EDI* and *HQ* values revealed a minimal exposure of the adult population to those metals through roasted-meats consumption. However, *EDI* values of As in some roasted meats are above standard limits. Roast meats with wood showed higher levels of major and trace elements than meats roasted with coal. High exposures, in the long-term, may cause damage to health.

## 1. Introduction

According to the Food and Agriculture Organization (FAO) of the United Nations, the primary sources of meat are domesticated animal species, such as cattle, pigs, birds, sheep, and goats, respectively [1]. The content of major elements (Ca, Fe, Mg, K, Na, and P) and trace elements (Zn, Mn, Cu, As, Cd, Hg, Pb, and Se) in meats [2], lipids, carbohydrates, proteins, color, and texture depend on several aspects, such as the region in which the animal is raised, genetics, age, food, type of cut, and tissue [3,4].

The Commission of the European Communities stipulated the maximum allowed limit of As, Cd, and Pb in poultry, cattle, pork, and sheep muscle and offal [5]. Different methods are used to determine the ingested amount of food contaminants, water, and other liquids. International organizations such as the Joint FAO/WHO Expert Committee on Food Additives (JECFA) and the European Food Safety Authority (EFSA) recommends the use of health-based guidance values (HBGVs) as provisional tolerable weekly intake (PTWI) and benchmark dose lower confidence limit (BMDL) for metals and metalloids considered contaminants that may accumulate in the body [6,7,8]. Other approaches to estimate the possible exposure to food additives from a diet comprise the estimated daily intake (*EDI*) [9] and the hazard quotient (HQ) method. The *EDI* is the amount of an additive ingested by the average consumer of the food [10,11], and the *HQ* is the ratio of the potential exposure to a substance at a level at which no adverse effects are expected, respectively [12]. Moreover, the minimal risk level (MRL) is an estimate of the daily human exposure to a hazardous substance that contains elements such as Al, Cd, Co, Cr, Cu, Mo, V, and Zn that is likely to be without appreciable risk of adverse [13]. On the other hand, for some elements, such as Cu, Fe, Mg, Mn, Mo, Ni, V, and Zn, there is a maximum level of daily nutrient intake (UL) that aims to avoid the risks of toxicity [14].

The way meats of different types are prepared [15], and especially storage conditions and handling, are potential sources of contamination and can significantly alter the levels of metals present in food [16], by placing food in contact with the contaminants. During the thermal processing of food, reactions that change color and taste occur, including the ones that result in toxic and mutagenic substances, such as furan, acrylamide, and acrolein [17]. Besides, fuels such as coal, which is used in barbecue grills, release heavy metals [18], becoming a possible food pollutant.

According to studies, metals are an expressive route of exposure to toxic compounds [19,20,21]. Total and bioaccessible concentrations of trace elements (Al, As, Cd, Cu, Fe, Hg, Mn, Ni, Pb, and Zn) were measured in charcoals from 15 barbecue products available from UK retailers [19]. According to an estimate of daily intake for the bioaccessible trace elements in the coal samples available in the United Kingdom, As and Al are the elements that caused the most significant concern of impact on human health [19]. The Canadian government considers charcoal as a source of hazardous emissions for health; thus, its sale and advertising are under regulation [20].

In many countries, like Brazil, Argentina, Paraguay, and Bolivia, the use of wood on a barbecue grill for roasting meat is a cultural issue. In Brazil, along with the various types of wood sold as barbecue fuels, some people use the remains of construction wood, without knowing that a number of them have undergone chemical treatment, such as *Eucalyptus* and other wood that is treated with Chromed Copper Arsenate (CCA) [22]. Consequently, wood smoke contains several toxic, harmful air pollutants, including As and Cr [23,24,25]. In view of the above, studies considering the *EDI* and comparisons with reference values for each element (PTWI, UL, BMDL, and MRL) can provide an overview of human-health-risk assessment [6,7,8,9,10,11,12,13,14].

Some studies reported the concentration of toxic and essential elements in several commercial types of meats, using different thermal preparation methods (roasting, boiling, and microwave cooking) [26], determination of metal in raw meats by inductively coupled plasma optical spectrometry (ICP-OES) [27] and inductively coupled plasma mass spectrometry (ICP-MS) [28], the role of detergent washing on levels of metals in meat [29], and validation of mineralization procedures for the determination of contents in raw meats samples by ICP-MS [30]. However, there is limited data on the major and trace elements composition of roast meats consumed by the public in Brazil and other countries, especially regarding the metal and metalloids content after barbecuing-smoke exposure. To date, there are no studies that have quantified the accumulation of macro- and microelements in different types of meat when roasted on barbecue grills, using different kinds of wood and charcoal as fuels. The aim of this study was (i) to determine the levels of Cd, Co, Cr, Mn, Mg, Cu, Al, Mo, Ni, Pb, V, Fe, Zn, and As in beef topside, pork loin, chicken breast, and lamb shank roasted on barbecues, using different types of fuels, such as woods and charcoal; (ii) obtain the *EDI* and *HQ* due to exposure to metals consumed from roasted meats, using wood and coals; and (iii) compare the *EDI* values in roasted meats with the values established by the PTWI, UL, BMDL, and MRL.

## 2. Materials and Methods

### 2.1. Sample Collection

Three samples of each type of raw meat were purchased from six different butcheries of Campo Grande, MS, in the Midwest region of Brazil, while considering the different origins in terms of the production system (meat labels) and anatomical location: beef (topside), pork (loin), lamb (shank), and chicken (breast without skin).

The following woods were acquired through direct purchase in commercial establishments in Campo Grande, Brazil: *Eucalyptus citriodora* wood, *Guazuma ulmifolia* wood, *Anadenanthera falcata* wood, and treated *Eucalyptus* (CCA-treated *Eucalyptus* wood). Two charcoals were purchased: *Eucalyptus citriodora* coal and *Guazuma ulmifolia* coal. The purchase of wood and coals were in 2019.

### 2.2. Preparation of Raw Meat Samples

Triplicate portions of proximate to 50 g of each meat sample were obtained by using stainless steel scalpels, individually, to avoid contamination. All samples were ground in a domestic processor with stainless steel blades (Thermomix TM5 equipment—Vorwerk L.L.C., Wuppertal, Germany) and homogenized to secure faithful batches of each type of meat. The samples were individually placed in universal sterilized plastic collectors previously identified and stored in a freezer at −20 °C until the time of analysis.

### 2.3. Preparation of Raw Meat Samples for Barbecue Grills

Slices proximate to 90 g in triplicate of three different samples of raw meat with 1.70 cm thickness were made, using stainless steel scalpels. Samples were divided into three groups: raw samples, samples roasted on a barbecue with wood or charcoal as fuels, and samples of hamburger roasted on an electric barbecue. The whole process of possible roasting combinations of the various meat samples on the masonry barbecue grill, using different types of wood and coals, was tested in triplicate and according to Table 1.

A masonry barbecue that was 60 cm long, 37 wide, and 31 cm deep was used to roast the meat. The wood or charcoal was evenly distributed inside the masonry barbecue. Meat samples were placed on a stainless steel grid, at the height of 40 cm from the wood or charcoal. Each sample of meat on the masonry barbecue grill remained for 30 min in the presence of firewood or charcoal, until roasting to the point. After the meat was roasted, samples were sliced, using a scalpel with stainless steel blades. Then, the samples were weighed and homogenized (Thermomix TM5 equipment—Vorwerk L.L.C., Wuppertal, Germany), and then stored in a universal plastic collector and frozen at −20 °C until analysis.

The following procedures were performed for hamburger samples roasted on an electric barbecue: Before the start of the cooking experiment, the electric barbecue grill was washed with detergent, rinsed thoroughly, and dried. Burgers of proximate to 50 g of four different samples of raw meat previously processed were made. The meat was heated by using an electric barbecue grill (Electric Barbecue Mister Grill Plus equipment—Cotherm LTDA, São Paulo, Brazil), at its maximum setting. After fifteen minutes of grilling, the meat was flipped, using a disposable plastic spoon, and grilling continued for fifteen more minutes, for a total of 30 min of grilling. The electric barbecue grill was switched off, and it was found that the meat was cooked in the center and roasted to the point. After the burgers were roasted, samples were sliced, using a scalpel with a stainless steel blade. Then, they were weighed, homogenized (Thermomix TM5 equipment—Vorwerk L.L.C., Wuppertal, Germany), and stored in a universal plastic collector and taken for analysis. Experiments were performed in triplicate.

### 2.4. Preparation of Raw and Roasted Meat by Microwave Digestion

A 300 mg mass of each sample was cut with stainless steel scalpels and then weighed (raw meat and roasted meat) in the digestion vessels. We added 2 mL of HNO_3_ (65% Merck, Darmstadt, Germany), 1.5 mL of H_2_O_2_ (30% Merck, Darmstadt, Germany), and 2 mL of ultrapure deionized water (18 MΩcm, Milli-Q Millipore, Bedford, MA, USA) to each sample. The samples were digested in a microwave digestion system (Speedwave four^®^, Berghof, Germany). This application used the temperature program shown in Table 2. The samples were then transferred to clean a polyethylene tube and diluted with ultrapure water to 10 mL. All digestions were performed in triplicate for fresh and roasted meat samples and analytical blanks.

### 2.5. Elemental Measurement by Using ICP-OES

The determination of major and trace elements in samples of different types of raw and roasted meat was performed by using an inductively coupled plasma optical emission spectrometer (ICP-OES) with an axial view (iCAP 6300 Series, Thermo Scientific, Waltham, MA, USA). The instrumental and operating parameters for ICP-OES are shown in Table 3.

An addition/recovery test for the elements under study was carried out in a meat sample by spiking (0.5 and 1.0 mg/L of each analyte). Table 4 shows that the method had a recovery interval of 90–111% for the spike of 0.5 mg/L and 93–112% for the spike of 1.0 mg/L. Thus, the recovery test shows that there were no systematic errors or losses of elements during the digestion process.

### 2.6. Calibration Procedure

The calibration curves for all the analytes were built on nine different concentrations over the range of 0.005–2 mg/L. A multielement solution containing 100 mg/L Al, Cu, Fe, Mg, Mn, Mo, Ni, Co, V, and Zn (SpecSol-Quinlab, São Paulo, Brazil) and a monoelementar solution containing 100 mg/L As, Cd, Cr, and Pb (SpecSol-Quinlab, São Paulo, Brazil) of each element were used to build calibration curves.

The calculation of the limits of detection (LOD) and limits of quantification (LOQ) was according to the analytical standards established by the IUPAC [31]. Table 5 shows the parameters of the calibration curve, as well as LOD and LOQ values and the obtained correlation coefficient (*R*^2^). The values of LOD were in the range 0.0002–0.0045 (mg/L) and the LOQ were 0.007–0.0151 (mg/L).

### 2.7. Human Health Risk Assessment

The method used in this study was for non-carcinogenic and adapted from the method described by Onsanit et al. [9]. The human health risk was assessed by considering the *EDI* for a chemical contaminant in meats, as well as the intake amount of the meats. It is possible to calculate the *EDI* (µg/kg bw/day) through consumption of food, using the following equation:

(1) $$EDI=C_{meat}x\left[\frac{dc_{meat}}{bw}\right]$$

where, *C_meat_*, *dc_meat_,* and *bw* (bodyweight) represent heavy metal content in raw and roasted meats (µg/g), daily meat consumption per capita (g/day), and the adult’s body weight. The average weight of a Brazilian adult is 70 kg, and the average daily consumption of beef, pork, poultry, and lamb in Brazil is 63.2, 8.5, 36.5, and 0.8 g/person/day, respectively [32]. Other than that, the risk to human health by intake of heavy-metal-contaminated meats was characterized by using a hazard quotient (*HQ*). The *HQ* is a ratio of *EDI* and chronic oral reference dose (*RfD*), which is determined by the following equation:

(2) $$HQ=\frac{EDI}{RfD}$$

The *RfD* values for the risk calculation were established by the Joint Food and Agriculture Organization/World Health Organization Expert Committee on Food Additives [6] and the United States Environmental Protection Agency (USEPA) [33]. The *RfD* values for the elements are as follows: Al = 0.4 µg/kg bw/day, As = 0.3 µg/kg bw/day, Cr = 3 µg/kg bw/day, Cu = not available, Fe = 700 µg/kg bw/day, Mn = 140 µg/kg bw/day, Mo = 5 µg/kg bw/day, V = 9 µg/kg bw/day, Mg = 11,000 µg/kg bw/day, Ni = 20 µg/kg bw/day, Cd = 1 µg/kg bw/day, Pb = 4 µg/kg bw/day, Co = 30 µg/kg bw/day, and Zn = 300 µg/kg bw/day [33]. In Equation (2), toxic risk is considered to occur if *HQ* > 1, while *HQ* < 1 represents negligible hazard (adverse non-carcinogenic effects).

### 2.8. Statistical Analysis

One-way ANOVA statistically analyzed differences between the groups, with post hoc Tukey’s test multiple comparison in GraphPad Prism 8.0 software. The significance level was set at *p* < 0.05.

## 3. Results and Discussion

In this section, the paper is organized as follows: Section 3.1 contains data on the concentration of major and trace elements obtained for raw and roasted meats, and the comparison of these values with studies carried out in other countries and published in the literature. In Section 3.2, we present the results obtained according to the type of elements quantified for each meat, since we consider that this is one of the factors that influences the calculation of the *EDI*, which is a common index for the transfer of metal from meat to man.

### 3.1. The Concentration of Major and Trace Elements Obtained in Raw and Roasted Meats with Different Fuels

Table 6 shows the major element (Mg) and trace elements (Al, As, Cd, Cr, Cu, Fe, Mn, Mo, Ni, V, and Zn) obtained by using ICP-OES in four different raw meat types, in an electric grill, and also from the respective meats roasted with six different types of wood and coal as burning fuels. Further details of the statistical results obtained from comparisons between samples are available in Supplementary Materials Figure S1. The metals Co and Pb were below the limit of detection.

#### 3.1.1. Aluminum (Al)

Table 6 shows the Al content in raw and roasted topside beef in the electric grill and with different burning fuels. There is no statistical difference between the content of Al in raw meats and in meats roasted with *Eucalyptus citriodora* wood; however, the values of this element in meats ranged from 1.25 to 3.31 mg/kg. However, the concentration of Al is high in beef roasted with electric grill (9.78 ± 1.69 mg/kg), as well as in CCA-treated *Eucalyptus* (11.61 ± 0.60 mg/kg), *Anadenanthera falcata* wood (17.49 ± 0.23 mg/kg), and *Guazuma ulmifolia* coal (27.79 ± 0.71 mg/kg). In the pork samples, all treatments differed from the Al level in raw meat, but the values that most stood out were pork loin roasted with *Anadenanthera falcata* wood (15.33 ± 0.46 mg/kg), electric grill (25.53 ± 0.21 mg/kg), and *Guazuma ulmifolia* coal (56.90 ± 0.73 mg/kg). The Al concentration in lamb shank portions roasted with *Eucalyptus citriodora* wood (2.89 ± 0.05 mg/kg) did not diverge from the raw lamb sample (1.54 ± 0.55 mg/kg). On the other hand, it exhibited intermediary levels after the treatment with *Anadenanthera falcata* wood, *Guazuma ulmifolia* wood, CCA-treated *Eucalyptus* and electric grill (12.12 ± 0.64–17.18 ± 1.92 mg/kg). In addition, higher levels of Al were found in lamb meat after roasting it with *Guazuma ulmifolia* coal 28.22 ± 0.64 mg/kg. The highest levels of Al in chicken breast meats appeared after roasting with *Guazuma ulmifolia* coal (46.46 ± 1.03 mg/kg), followed by electric grill (37.30 ± 0.19 mg/kg), and *Anadenanthera falcata* wood (23.12 ± 0.86 mg/kg). All of these treatments differed from the raw chicken meat, except the chicken roasted with *Eucalyptus citriodora* wood. Thus, the Al concentrations in some groups are statistically different.

In Table 6, the content of Al in raw portions of meat as beef topside (1.25 ± 0.23 mg/kg), pork loin (0.86 ± 0.12 mg/kg), chicken breast (1.01 ± 0.15 mg/kg), and lamb shank (1.54 ± 0.55 mg/kg) is lower than those published in the literature in the Canary Islands by González-Weller et al. [34] for fresh cuts of chicken breast (9.12 ± 1.65 mg/kg), pork (9.78 ± 5.14 mg/kg), and beef (8.74 ± 4.72 mg/kg), respectively. On the other hand, the Al concentration in our results and those quantified by González-Weller et al. [34] is higher than that found by Leblanc et al., for porcine muscle (0.21 mg/kg) and poultry (0.26 mg/kg) [35].

Notably, the roasting process with *Guazuma ulmifolia* coal affected all meat types, increasing the Al content in a significant manner, indicating that this kind of fuel may incorporate this metal to the meats through the smoking process.

#### 3.1.2. Arsenic (As)

In Table 6, the As concentration raised significantly in all meat cuts roasted with CCA-treated *Eucalyptus* regarding the raw and grilled samples. Considering that the CCA-treated *Eucalyptus* receives a treatment of CCA, this high amount in this particular fuel is expected [24].

The content of As in raw samples of beef topside, pork loin, and chicken breast is superior than the results obtained in a research carried out in Saudi Arabia for red meat 0.01 mg/kg and raw chicken 0.03 mg/kg [36], and those found in studies in Italy for raw equine meat 0.068 ± 0.005 mg/kg [37], and in Taiwan (raw beef = 0.008 ± 0.009 mg/kg and pork = 0.018 ± 0.027 mg/kg) [38]. Nonetheless, our results are in the range level of As in meat reported by FAO/WHO (0.004–0.78 mg/kg) [2].

#### 3.1.3. Cadmium (Cd)

Cd concentration was below the limit of detection in all raw and roasted samples of beef topside, pork loin, lamb shank, and chicken breast, using wood or charcoal as fuels (Table 6). With the exception of lamb shank, there was quantification of Cd in beef topside, pork loin, and chicken breast, using the electric grill, unlike in previous studies, where the Cd concentrations for raw and grilled meat remained the same or decreased (fresh > grilled) [15,39].

#### 3.1.4. Chromium (Cr)

Cr was not detected in the raw samples of pork, lamb, and chicken, while the beef was the only fresh meat that contained this metal (Table 6). In all samples, the Cr concentration was higher in the meats roasted with CCA-treated *Eucalyptus* [40] and the electric grill, diverging from the raw meat in all meat types, but for chicken roasted with the electric grill. It is known that stainless steel equipment can contain Cr, to improve the corrosion resistance [41,42]. In fact, as we can see in Table 6, higher levels of Cr are quantified from using the electric grill and CCA wood. Thus, both CCA wood and the stainless steel barbecue may be the possible cause of contamination of Cr in the meat. Moreover, Cr content in raw beef topside was 0.15 ± 0.24 mg/kg, which is less than the values quantified in Saudi Arabia for raw red meat (0.25 mg/kg) [36], and Nigeria fresh bovine muscle (1.24 ± 0.52 mg/kg) [43].

#### 3.1.5. Copper (Cu)

In Table 6, the Cu content for all roasted beef varied around 0.29 to 2.31 mg/k, showing a statistically significant difference in relation to the raw sample. The Cu content in pork samples registered significant differences when using *Guazuma ulmifolia* coal (0.33 ± 0.12 mg/kg), electric grill (0.53 ± 0.01 mg/kg), and CCA-treated *Eucalyptus* (0.71 ± 0.16 mg/kg). The Cu content in raw lamb (0.84 ± 0.01 mg/kg) differed from lamb roasted with the electric grill (1.95 ± 0.12 mg/kg), *Guazuma ulmifolia* wood (1.29 ± 0.07 mg/kg), *Eucalyptus citriodora* coal (1.97 ± 0.24 mg/kg), and *Guazuma ulmifolia* coal (2.10 ± 0.09 mg/kg). Moreover, higher levels of Cu occur in lambs roasted with CCA-treated *Eucalyptus* (2.80 ± 0.02 mg/kg). As noted in other samples, the Cu levels in raw chicken show statistically significant differences in comparison with CCA-treated Eucalyptus samples (0.63 ± 0.22 mg/kg). In fact, the presence of elements such as Cu in roasted meats can be explained due to the CCA-treated eucalyptus wood [24,40], composition of the fuel itself [20], and the contamination of the electric grill [41,42,44].

The values of Cu concentration in raw beef topside (0.29 ± 0.03 mg/kg) (Table 6) are below the values found in raw beef in Zurich (0.498 ± 0.279 mg/kg) [28] and Saudi Arabia (14.84 ± 0.40 mg/kg) [45], as well as in muscle (34.8± 15.6 mg/kg), liver (22.3 ± 11.1 mg/kg), kidney (20.4 ± 5.9 mg/kg), and heart 18.8 ± 4.1 mg/kg of bovine collected from Egypt [29]. In Table 6, the concentration of Cu in raw lamb shank (0.84 ± 0.01 mg/kg) was below than that found in the USA among lamb muscle groups with different origins, which varied from 1.30 ± 0.04 to 0.94 ± 0.03 mg/kg [46].

#### 3.1.6. Iron (Fe)

The Fe concentration changed considerably with kind of fuel used (Table 6). Highest Fe values obtained were in beef samples roasted with the *Eucalyptus citriodora* coal (82.03 ± 16.08 mg/kg) and *Guazuma ulmifolia* coal (87.08 ± 0.51 mg/kg). The levels of Fe in pork samples varied significantly for all fuels regarding the raw meat. However, roasting the pork in the electric grill (8.52 ± 0.52 mg/kg), *Eucalyptus citriodora* (8.13 ± 0.24 mg/kg), *Anadenanthera falcata* (12.06 ± 0.50 mg/kg), and *Guazuma ulmifolia* (11.68 ± 0.81 mg/kg) woods did produce a difference with the raw samples. Moreover, the Fe levels in lamb varied around 18.76–71.71 mg/kg, with a significant difference for all roasted lamb samples, when compared to the raw, but for the one roasted in the electric grill. Likewise, Fe levels in chicken roasted with *Anadenanthera falcata* wood (15.65 ± 0.54 mg/kg) were higher than that prepared with CCA-treated *Eucalyptus* (10.74 ± 0.28 mg/kg), but lower when compared to the *Guazuma ulmifolia* coal (19.35 ± 2.15 mg/kg). The increase in Fe content in all roasted meats may be partially related to the loss of water during the roasting process [47], or deposition of particles and gases from the wood or charcoal used [19,20,21,22,23,24,25].

Fe in raw beef topside (37.52 ± 1.58 mg/kg) is higher than raw beef (16 ± 2 to 25 ± 8 mg/kg) obtained in Zurich [28], and close to the value obtained by other Brazilian studies for beef (48 ± 2 mg/kg) [27]. On the other hand, the Fe content in pork loin (5.18 ± 0.26 mg/kg), chicken breast (4.58 ± 0.76 mg/kg), and lamb shank (18.76 ± 0.19 mg/kg) is lower than the content obtained in pork loin (7 ± 6 mg/kg), chicken breast without skin (5 ± 2 mg/kg), and lamb chop (20 ± 7 mg/kg) quantified in Zurich [28].

#### 3.1.7. Magnesium (Mg)

Mg levels (Table 6) in beef augmented significantly after roasting with coals and woods; however, there were lower concentrations in the beef roasted in the electric grill (267.62 ± 2.95 mg/kg) and *Guazuma ulmifolia* wood (307.96 ± 2.89 mg/kg). In the present study, Mg levels in raw pork loin varied from the roasted samples, with a high concentration in *Eucalyptus citriodora* coal (366.53 ± 24.81 mg/kg) and *Guazuma ulmifolia* wood (354.44 ± 3.91 mg/kg). The Mg content in lamb samples was higher after roasting with different fuels, especially after the use of *Guazuma ulmifolia* wood (359.58 ± 13.99 mg/kg). Likewise, Mg levels were high in chicken breast roasted with *Guazuma ulmifolia* wood (418.78 ± 12.02 mg/kg), CCA-treated *Eucalyptus* (414.27 ± 1.44 mg/kg), *Guazuma ulmifolia* coal (412.22 ± 0.04 mg/kg), and *Anadenanthera falcata* wood (407.88 ± 3.07 mg/kg).

Table 6 also shows that the concentration of Mg in raw beef topside, pork loin, chicken breast, and lamb shank were 218.47 ± 3.95 mg/kg, 225.55 ± 8.08 mg/kg, 291.95 ± 86.87 mg/kg, and 206.77 ± 3.99 mg/kg. Another Brazilian study carried out with bovine meat found that the Mg concentration was 700 ± 28 mg/kg [27]. In Romania, according to results, the Mg level in pork loin was 40.511 ± 1.62 mg/kg [26]. Studies carried out by the company Thermo Fisher Scientific, Cambridge, UK, using atomic flame absorption, obtained an amount of 19.579 mg/kg in chicken meat [48]. In Southern Italy, the mean values of Mg found in raw lamb meat samples ranged between 25.80 ± 5.67 and 37.24 ± 2.27 mg/kg [49]. When comparing Mg levels presented in Table 6 with those obtained in other studies [26,27,48,49], we found that metal concentrations in the meat cuts that we reported are far superior.

#### 3.1.8. Manganese (Mn)

However, after the meats were roasted using different fuels, it was observed that the values of Mn concentrations were higher for roasted meats when we used *Eucalyptus citriodora* charcoal and firewood. In other words, the results demonstrate that wood and/or coal can accumulate Mn, causing the contamination of the meat through the emitted smoke. Except for the chicken breast, this pattern is correct for all other meat types. However, even the chicken breast did not show an elevated amount of Mn when roasted with *Eucalyptus citriodora* coal; the highest Mn concentration happens with *Eucalyptus citriodora* wood treatment. While we did not detect Mn in raw meats, results obtained in Zurich obtained Mn values in beef ranging from 31 to 108 mg/kg, while, in pork meat, it varied from 62 to 128 mg/kg; for chicken breast without skin, the concentration of Mn was 79 mg/kg, and for lamb chop, the content of Mn was 167 mg/kg [28]. According to Gerber et al. [28], Mn concentrations vary unsystematically within muscles and species.

#### 3.1.9. Molybdenum (Mo)

In Table 6, with the exception of raw lamb shank (0.45 ± 0.05 mg/kg), the concentration of Mo in other raw meat is below the detection limit, as well as in samples roasted with most fuels. There was a statistical difference in Mo concentrations only in the sample of beef roasted with *Anadenanthera falcata* wood (0.55 ± 0.00 mg/kg), *Guazuma ulmifolia* wood (0.58 ± 0.01 mg/kg) and *Eucalyptus citriodora* wood (0.73 ± 0.01 mg/kg). Molybdenum levels in all pork loin and chicken breast samples were below the analysis method detection limit, as the samples of roasted lamb with *Eucalyptus citriodora* wood, *Guazuma ulmifolia* wood, and *Anadenanthera falcata* wood. Therefore, the values of raw lamb and roasted lamb with CCA-treated *Eucalyptus* were superior to the latter but lower than the quantities of Mo in roasted lamb with the electric grill (1.00 ± 0.04 mg/kg) and both types of coal.

#### 3.1.10. Nickel (Ni)

The Ni concentration in the samples of raw meat and meat roasted with wood and charcoal are below the detection limit (Table 6). However, the detection of Ni occurred after cooking the meat types in the electric grill, suggesting contamination from the grill material. Higher concentrations of Cr and Ni have been recognized to improve the corrosion resistance of the grill. In addition, the literature data are conflicting due to differences between the materials used in each country [42]. According to a survey on the metal composition of kitchen grids, kitchen grids can be coated with enamel, to protect against corrosion and to facilitate cleaning. However, when heated, they can emit As, Pb, Cd, Cr, Fe, Co, Li, and Ni [50]. Therefore, it is necessary to emphasize the importance of the quality of the product that may have influenced these results [41].

#### 3.1.11. Vanadium (V)

The V concentrations in the raw meats and roasted are shown in Table 6. There were statistical differences in roasted beef, pork, and chicken samples. For beef, the V values increased mostly in the samples roasted with *Eucalyptus citriodora* coal (1.02 ± 0.06 mg/kg) and *Guazuma ulmifolia* coal (1.08 ± 0.09 mg/kg); the same happened in pork samples.

The samples of lambs raw or roasted with any type of fuel did not differ significantly between them. On the other hand, the highest levels of V in chicken were found in samples roasted with *Guazuma ulmifolia* wood (13.82 ± 1.06 mg/kg) and charcoal (16.29 ± 0.40 mg/kg).

According to studies carried out in Yugoslavia, the concentration of V in steaks, pork leg, and chicken breast was 0.4 × 10^−^^3^ mg/kg, 0.6 × 10^−^^3^ mg/kg, and 1.7 × 10^−2^ mg/kg [51]. In South Africa, the concentration of V in bovine muscle was 0.325 ± 2 × 10^−3^ mg/kg [52]. In Hong Kong, the concentration of V in beef, mutton, pork, and chicken meat was 1.5 × 10^−3^ mg/kg for all samples [53]. In the USA, the concentrations of V in lamb muscles from different sources ranged from 0.25 ± 0.18 to 2.3 ± 1.4 mg/kg. Thus, it is observed that the values quantified in the meat of Table 6 are higher than those of the data found in Yugoslavia [51], South Africa [52], Hong Kong [53], and the USA [47].

#### 3.1.12. Zinc (Zn)

In comparison with raw meats, it was observed that there was an increase in the concentration of Zn in all roasted meats, using different fuels (Table 6). In fact, variations in the concentration values of some elements such as Fe and Zn in meat samples can occur due to water loss during the cooking process [28]. However, the treatment with *Guazuma ulmifolia* coal elevated the Zn quantities the most, except for the lamb shank, which presented a higher Zn concentration when roasted with *Guazuma ulmifolia* wood. Again, these results imply that *Guazuma ulmifolia* may have an elevated amount of Zn in its composition and that it would be transferred to the food through the smoke.

The results obtained in our study are lower than those obtained in France for the concentration of Zn in pork (36.76 mg/kg) and chicken muscle 16.23 mg/kg [35], and cow muscle from Nigeria (121.27 ± 7.45 mg/kg) [43]. In contrast, data concerning the concentration of Zn in seven chicken samples from different areas of Nigeria ranged from 6.12 ± 0.15 to 33.21 ± 43.34 mg/kg [54]. Meanwhile, Zn concentrations in Morocco for cattle and sheep meats were 9.04 ± 0.17 and 6.97 ± 0.17 mg/kg, respectively. Thus, the zinc concentration values in Table 6 are close to the minimum values obtained in Nigeria for chicken samples. Moreover, the concentration of Zn in raw beef topside and raw lamb shank is higher than in Morocco for cattle meat had and sheep meat [54,55].

#### 3.1.13. Metal and Metalloid Content Regarding Meat Types and Relationship with the Fuel

The result of the present study showed that meat roasted with charcoal has a lower level of trace elements when compared to meat roasted with firewood. Mg has reported the highest level, while Al, Fe, Cr, Cd, Ni, and As were the lowest in all meat types. Moreover, all roasted meat with wood fire from CCA-treated *Eucalyptus* presented high levels of As, Cu, and Cr, which can relate to the presence of these elements in the wood treated with CCA [22,23,24,25]. Although this kind of wood is often used as a burning fuel to barbecue, the presence of toxic metals and metalloids, such As, and subsequent release and impregnation of this contaminant in food through smoke make its use unsafe. In the meat types roasted with coal, the level of As, Cu, and Cr were lower than in roasted meat with wood treated with CCA (Table 6).

The combustion of coal releases particles that contaminate the meat with amounts that cannot be neglected [20]; for some elements, such as Al, the sum of the elemental deposition from fuel increased the initial concentration in over 37-fold. Furthermore, previous research investigating a total of 24 quantified elements (Al, Ag, As, Be, Bi, Ca, Ca, Ce, Co, Cr, Cs, Cu, Fe, La, Mg, Mn, Mo, Ni, Pb, Rb, Se, Sr, V, and Zn) associated the inhaling of the elemental fine airborne particles released during charcoal combustion with health hazards. Moreover, changes in fatty acids concentration during the grilling of meat, as told by Rogge et al. [56], led to the higher production of aerosols made of fatty acids from oil or grease droplets that fall into the heat source, where they would vaporize and renucleate and grow into small particles; considering the distance between the meat and the heat source, in the case of electric grills and other types of portable grills, it would be an aggravating factor. The elements As, Cr, Se, V, and Zn were the most abundant metals identified in the fine particles [21]. Studies on daily exposure of a grill operator, while grilling meat [19], and the potential risk associated with the use of BBQ charcoal [20,21], indicate that the lifetime risk of cancer associated with exposure to As and Cr is significant in restaurants [21], and in domestic use, which becomes a public health problem.

In the case of enameled steel and cast-iron grills used in grills, the Federal Institute for Risk Assessment (*BfR*) examined whether and to what extent the metallic elements of the enamel layer of the grates are released during cooking and can therefore pass for grilled foods. It was found that, in some materials of the examined grids, considerable amounts of aluminum, antimony, arsenic, and nickel escape. The *BfR* assessed the integrity of the results and concluded that the health-tolerable exposure values are sometimes significantly exceeded [50].

### 3.2. Estimated Daily Intake (EDI) through Meats Consumption

*EDI* and *HQ* for individual elements caused by the intake of different roasted-meat types with different fuels are estimated in Table 7 and Table 8. The *EDI* and *HQ* of elements through roasted beef topside and roasted pork loin consumption in Brazil are presented in Table 7, and for the elements due to the consumption of roasted chicken breast and roasted lamb shank are in Table 8. The *EDI* of all studied major and trace elements was calculated based on mean meats consumption in Brazil (g/person/day).

#### 3.2.1. Aluminum (Al)

The *EDI* of Al due to intake for roasted beef topside with electric grill, wood fire, and coals ranged from 2.9885 to 25.2078 µg/kg bw/day. For roasted pork loin with electric grill, wood fire and coals, the *EDI* of Al due to intake of this meat ranged from 0.3461 to 6.9093 µg/kg bw/day (Table 7). The *EDI* of Al through roast chicken breast meat consumption ranged from 0.9438 to 24.2256 µg/kg bw/day, and roasted lamb shank ranged from 0.0330 to 0.3225 µg/kg bw/day (Table 8). The provisional tolerable daily intake (PTDI) suggested by the JECFA for Al is 285 µg/kg bw/day [57]. On the other hand, the MRL of 1000 µg Al/kg/day has been derived for intermediate-duration oral exposure (15–364 days) to Al [13]. Therefore, *EDI* values of Al are below the exposure limit of the JECFA and values of the MRL, when considering the consumption of roasted meats only.

In Table 7, except for the roasted pork loin with wood fire from *Eucalyptus citriodora*, beef topside, and pork loin, both roasted with other wood and coal presented *HQ* values of Al for adults superior to 1. Besides, in Table 8, samples of roasted chicken breast with wood fire and coal had *HQ* values for Al above 1 for adults. That is, the results show that, in the exposed population, chronic health risks may occur. On the other hand, roasted lamb shank with wood fire and coal showed *HQ* values of Al for adults below 1, indicating that the consumption of this roasted meat may not cause adverse effects to the health of the population that consumes it. It is critical to highlight that those values are considering only meat consumption and not the smoke exposure or other contaminations sources, which would add to that, worsening the problem. According to studies, people exposed to high levels of Al may develop neurological impairments, such as Alzheimer’s disease [58,59].

#### 3.2.2. Arsenic (As)

*EDI* of As values due to consumption of roasted beef meat with electric grill, wood, and coals varied from 0.2618 to 18.9961 µg/kg bw/day, while the roasted pork loin with electric grill, wood fire, and coal ranged from 0.0231 to 1.8336 µg/kg bw/day (Table 7). Moreover, the *EDI* of As due to intake of roasted chicken breast meat consumption ranged from 0.1929 to 13.6875 µg/kg bw/day, and roast lamb shank *EDI* values were between 0.0011 and 0.2013 µg/kg bw/day (Table 8).

Since the PTWI established by JEFCA for As was considered no longer appropriate, the risk assessment was performed by using the BMDL_01_ that the EFSA has established to estimate the dietary risk, ranging values between 0.3 and 8 µg/kg bw/day for cancers of the lung, bladder, and skin [7,60]. On the other hand, the ATSDR has established an MRL for As of 0.3 μg/kg bw/day for chronic duration exposure (≥1 year) [13]. *EDI* values for As in some roasted meats, compared with those proposed by both EFSA and JECFA, demonstrated that *EDI* values are above this limit.

Table 7 shows that the concentration of As for the roasted beef meat with wood fire from *Guazuma ulmifolia* and *Anadenanthera falcata*, where the values of *HQ* are inferior to 1. However, the other samples of roast beef meat with electric grill, wood fire, and coal presented values above 1. Only the roasted pork loin with wood fire from CCA-treated *Eucalyptus* had values *HQ* of As for adults greater than 1. In the same way, *HQ* of As for adults is greater than 1 for roasted chicken breast with coal fire from *Eucalyptus citriodora*, roasted chicken breast with coal fire from *Guazuma ulmifolia*, and roasted chicken breast with wood fire from CCA-treated *Eucalyptus*. All other samples have *HQ* reference values lower than 1, meaning that no adverse effects are expected, while *HQ* above 1 can be a concern regarding adverse non-carcinogenic effects.

As is toxic in its inorganic and organic forms. Long-term exposure to As, mainly through cereal grains and food [61], can lead to chronic As poisoning [62]. Thus, the possibility of risk to some consumers is patent.

#### 3.2.3. Cadmium (Cd)

The Cd *EDI* concentration in Table 7 and Table 8 was below the limit of detection in all raw and roasted samples of beef, pork, lamb, and chicken, except for the meat cuts cooked on the electric grill. However, the *HQ* values of Cd for adults in electric-grill-roasted meats were still less than 1. Moreover, the values of the MRL derived for intermediate-duration (15–364 days) and chronic (1 year or longer) oral exposure for Cd is 0.5 µg/kg bw/day and 0.1 µg/kg bw/day, respectively [13]. Thus, the results obtained for Cd in electric grill are within this limit.

#### 3.2.4. Chromium (Cr)

The *EDI* of Cr due to intake of roasted beef and pork with wood fire and coals ranged from 0.0036 to 0.9931 µg/kg bw/day and 0.0097 to 0.0959 µg/kg bw/day, respectively (Table 7). Meanwhile, the *EDI* for Cr in roasted chicken breast ranged from 0.0521 to 0.4641 µg/kg bw/day, and for lamb shank from 0.0003 to 0.0103 µg/kg bw/day, correspondingly. There is no suggested value of PTDI determined by JECFA for Cr. However, the MRL of 5 µg/kg bw/day was derived for intermediate-duration oral exposure (15–364 days) to Cr [13]. Therefore, our results are within the limits established by the MRL.

In Table 7 and Table 8, the *HQ* values of Cr for adults in all roasted meats were less than 1. There are potential effects caused by Cr unbalance in adults, such as bronchial asthma, lung, and nasal ulcers and cancers, skin allergies, reproductive and developmental problems, and carcinogenic [63]. With the *HQ* results below 1, the consumption of these preparations may fall into the other side of Cr intake, being an essential nutrient for regular protein, fat, and carbohydrate metabolism [64] and critical to health and nutrition.

#### 3.2.5. Copper (Cu)

The values of the *EDI* of Cu due to the ingestion of roasted beef, pork, lamb, and chicken (Table 7 and Table 8) with electric grill, wood, and coals varied between 0.8487 and 2.0856 µg/kg bw/day, 0.0158 to 0.0862 µg/kg bw/day, 0.0469 and 0.3285 µg/kg bw/day, and 0.0095 and 0.0320 µg/kg bw/day, respectively. According to the JECFA, the value of PTDI suggested for Cu is 500 µg/kg bw/day, and the values of the MRL derived for intermediate-duration oral exposure (15–364 days) for the Cu is 10 µg/kg/day [13,65]. Thus, the *EDI* values of Cu in all samples of meat were below the permissible limits (PTDI and MRL). Therefore, the consumption of one portion of meats roasted does not confer a risk of adverse health effects for adults. However, the continued ingestion of this roasted meat can cause toxicity. Increased meat-eating increases Cu absorption and overall Cu exposure. Cu intake is a significant risk factor for Alzheimer’s disease [66]. Nonetheless, when consumed within the safe limits, Cu plays several roles in human metabolism, acting as a cofactor and structural constituent of enzymes [67].

#### 3.2.6. Iron (Fe)

Concerning the roast beef meat with electric grill, wood, and coals, the value of *EDI* of Fe ranged from 30.4534 to 78.6208 µg/kg bw/day. The *EDI* of Fe due to consumption of roasted pork loin with electric grill, wood fire, and coals ranged from 0.9872 to 1.4644 µg/kg bw/day (Table 7). The intake of chicken breast with electric grill, wood fire, and coals provided *EDI* values for Fe from 3.6083 to 10.0896 µg/kg bw/day. Moreover, the *EDI* of Fe due to intake for roasted lamb shank with electric grill, wood fire, and coals varied from 0.3935 to 0.8195 µg/kg bw/day (Table 8). The value of PTDI of Fe established by the JECFA is 800 µg/kg bw/day [68], but there are no values of the MRL set for Fe. From a comparison of the values *EDI* with those proposed by PTDI, it is possible to conclude that the *EDI* value for Fe is below the PTDI value. The high consumption of red meat is the leading cause of the increase in the risk of non-communicable diseases, type II diabetes, and cardiovascular disease. This hazard could be related to Fe intake [69,70]. However, the *HQ* of Fe for adults in all roast meats types was less than 1 (Table 7 and Table 8). In this way, roasted meats do not represent a risk for adverse health effects for adults. Sufficient data to define a safe lower limit for toxic Fe ingestions are not available. Fe deficiency is the most prevalent worldwide [71]; therefore, the consumption of appropriate amounts of this metal in consort with a balanced diet is recommended.

#### 3.2.7. Magnesium (Mg)

The values of the *EDI* of Mg due to the ingestion of roasted beef meat with electric grill, wood, and coals varied in a range from 278.0439 to 334.2648 µg/kg bw/day. *EDI* values due to the consumption of roasted pork loin with electric grill, wood fire, and coals varied from 24.9888 to 44.5072 µg/kg bw/day (Table 7). The observed range of *EDI* of Mg in the current study for roasted chicken breast with electric grill, wood fire, and coals is between 173.7921 and 218.3639 µg/kg bw/day. Moreover, the *EDI* of Mg due to intake for roasted lamb shank with electric grill, wood fire, and coals varied from 3.2890 to 4.1095 µg/kg bw/day (Table 8). There are no values established by the JECFA (PTDI) and the MRL for Mg. However, the UL level of Mg for males/females (19–70 years old) is 350 mg/day [14]. The tolerable upper intake level is the highest level of daily nutrient intake that is likely to pose no risk of adverse health effects in almost all individuals. Thus, the values *EDI* of Mg in all samples of meat were below the permissible limits by UL.

The *HQ* values of Mg for adults in all roasted-meat-types consumption for adults were less than 1 (Table 7 and Table 8). Therefore, the presence of Mg in meat does not cause any harm to health. To date, there are no studies of Mg intoxication due to food intake. However, substantial doses of Mg-containing laxatives and antacids have been associated with Mg toxicity because of excessive oral consumption [72].

#### 3.2.8. Manganese (Mn)

The values of the *EDI* of Mn due to the ingestion of roasted beef, pork, lamb, and chicken with electric grill, wood, and coals ranged from 0.0036 to 0.08667 µg/kg bw/day, 0.0024 to 0.1214 µg/kg bw/day, 0.0521 to 0.2868 µg/kg bw/day, and 0.0002 to 0.0063 µg/kg bw/day, respectively (Table 7 and Table 8). According to the JECFA, the value of PTDI suggested for Mn is 140 µg/kg bw/day [6]. There are no values of the MRL derived for oral exposure to the Mn. The *EDI* values of Mn in all samples of meat were below the established limit by PTDI, indicating a safe consumption with these treatments concerning this metal.

In all meats roasted with electric grill, wood and coal, Mn *HQ* values for adults were below 1 (Table 7 and Table 8); it represents negligible hazard when assessing hazards by severity, thus corroborating the findings of the EDI values. No evidence shows Mn toxicity from high dietary Mn intakes due to meat and other foods. However, people who consume 28 mg/L of water containing high levels of elements such as Mn can develop toxicity [73]. Although Mn is an essential nutrient, especially regarding the reduction of oxidative stress, both Mn deficiency or overload is rare, whereas the excess is usually caused by environmental exposure [74,75].

#### 3.2.9. Molybdenum (Mo)

The *EDI* of Mo due to the consumption of roasted beef meat with electric grill, wood fire, and coals and roasted lamb shank with wood and coals varied from 0.4966 to 0.6591 µg/kg bw/day and from 0.0077 to 0.010 µg/kg bw/day (Table 7 and Table 8). There are no values established by the JECFA (PTDI) for Mo. However, the MRL is 60 µg/kg/day [13]. Therefore, the *EDI* of Mo due to the ingestion of the roasted meat is below the MRL value.

The *HQ* of Mo for adults in all roasted meat types was inferior to 1. The toxicity of Mo compounds is low in humans. Currently, clinical data are scarce to allow definitive conclusions about the effects of Mo toxicity due to Mo supplementation and foods [76].

#### 3.2.10. Nickel (Ni)

The Ni *EDI* concentration was below the limit of detection in all raw and cooked samples of beef, pork, lamb, and chicken, except for the electric grill samples. For all roast-meat types in Table 7 and Table 8, the *HQ* values of Ni for adults were below 1, so roasted meats with these fuels do not represent a risk of adverse health effects for adults. A caveat is in relation to the stainless steel grill used during the preparation. Although this is not considered a dangerous material and has great application for hygiene and food safety, there are records of dermatitis resulting from Ni in the diet, as demonstrated in previous studies [77]. A dose of only 0.067 mg of Ni has been associated with cutaneous reactions in 40% of participants with Ni sensitivity [77,78]. Our result showed Ni values higher than those [77,78]; therefore, leached Ni can be relevant for highly sensitive patients.

#### 3.2.11. Vanadium (V)

The *EDI* of V in all meat types varied from 0.0009 to 0.9751 (Table 7 and Table 8). There is not a set value of PTDI for V established by the JECFA, but there are values of the MRL derived for intermediate-duration oral exposure, which is 100 µg/kg/day [13]. From the comparison, values *EDI* with those proposed by MRL, it is viable to conclude that the *EDI* value for V in Table 7 and Table 8 are below the MRL value.

For all roast-meat types in Table 7 and Table 8, the *HQ* values of V for adults were below 1, so roasted meats with these fuels do not represent a risk of adverse health effects for adults. Food and water are the primary sources of exposure to V for the general population. Symptoms of V toxicity vary with chemical form and route of absorption. Stomach cramps were reported in a study of people taking about 13 mg V/day [79], and its intake is not considered to be essential [80].

#### 3.2.12. Zinc (Zn)

The values of the *EDI* of Zn from the ingestion of roast beef meat with electric grill, wood, and coals varied in a range between 42.1905 and 104.0453 µg/kg bw/day. The *EDI*, due to the consumption of roasted pork loin with electric grill, wood fire, and coals, varied from 1.8251 to 2.5694 µg/kg bw/day (Table 7). The observed range of *EDI* of Zn in the current study for roasted chicken breast with electric grill, wood fire, and coals was found to be between 4.5521 and 8.4941 µg/kg bw/day. The *EDI* of Zn due to intake for roasted lamb shank with electric grill, wood fire, and coals varied from 0.5234 to 0.9273 µg/kg bw/day (Table 8). The value of PTDI established by the JECFA for Zn is 300 µg/kg bw/day [65], and the values of the MRL derived for intermediate-duration oral exposure (15–364 days) for the Zn is 300 µg/kg/day [13]. Thus, the *EDI* values of Zn in all samples of meat were below the permissible limits (PTDI and MRL).

The *HQ* values of Zn for adults are below 1 in all examples of roasted meat. The impact on human health by intoxication with Zn is a rare event, as Zn has low toxicity [81]. Excessive amounts of Zn in the body may cause harmful effects to the kidneys, liver, spleen, brain, and heart [82,83]; nonetheless, Zn essentiality in unquestionable, as Zn participates in cellular function, differentiation, division, and growth [84].

## 4. Conclusions

The present study showed that there are statistical differences in the contents of major (Mg) and trace elements (Al, As, Cr, Cu, Fe, Mn, Mo, V, and Zn) in raw meats, meats roasted with an electric grill with electric grill, and roasted meats on barbecues grill, using different fuels.

The concentrations of Al, Cr, Cu, and Fe in raw meats were below the values obtained by other countries. However, high levels of As in the raw beef topside, raw pork loin, raw chicken breast, and Mg, V, and Zn in all raw meats were quantified in this study. The content of Cu, Cr, and As were higher in meats roasted using wood. On the other hand, there was an increase in the values of V, Al, and Fe when the meat was roasted using charcoal.

The concentration of Ni and Cd was high when the meats were roasted using an electric grill. In fact, the material from which electric grills are made can contaminate meat. Mo levels were quantified in the raw and roasted leg of lamb shank with the electric grill, and in the roasted beef and leg of lamb shank, using different fuels. On the other hand, the variations in the concentrations of Mg, Mn, and Mo in the roasted meat are due to the presence of particles and gases from wood and coal. The levels of all measured elements in raw meats and in electric grilled meats are usually lower than those in the roasted meats with coal and wood, except for Ni and Cd.

There are no values established by MRL for Fe, Mg, Ni, and Mn. In addition, there is no value of PTDI suggested by JECFA for Cr, Mo, Mg, and V. The *EDI* of Al, Cu, and Zn are below the limit of JECFA and MRL reference levels. However, the *EDI* values of Cr, Mo, and V found in this study did not exceed the MRL standard. The *EDI* values of Fe and Mn are below the PTDI value. The *EDI* values of Mg in all samples of meat were below the permissible limits by UL. However, *EDI* values of As in some roasted meats are above of EFSA and JECFA limits.

Samples of roast chicken breast with wood fire and coal presented *HQ* values of Al and As for adults above 1. Moreover, *HQ* values of Cr, Cu, Fe, Mn, Mg, Mo, V, and Zn for adults in all roasted meats were less than 1. According to the Agency for Toxic Substances and Disease Registry, the effects of exposure to any hazardous substance depend on the dose and principally the duration.

Although *HQ* is less than 1, the health hazards associated with the use of woods and charcoal are of significant concern, and smoke associated with this meat-roasting process represents a significant source of contaminants and can add to the ingestion source. Levels of Cd and Ni were detected in the meats prepared in the electric grill, in opposition to the meats roasted with different types of woods and coals, which raises a concern on the day-to-day use of this equipment present in several households.

Finally, the metals and metalloids emitted by the combustion of coal or wood combustion in restaurants or domestic kitchens, wood stoves, and barbecue grills should be investigated, considering the risk to children, the elderly, and pregnant women, especially. High exposures to a long-term contaminant may cause damage to health.

## Figures and Tables

**Table 1:** Meat samples and types of fuels.

Meat Sample	Types of Fuels		
	Biomass		Electricity
	**Wood**	**Coal**	
Beef topside (*n* = 18)	*Eucalyptus citriodora*	*Eucalyptus citriodora*	Electric barbecue grill
Pork loin (*n* = 18)	*Guazuma ulmifolia*	*Guazuma ulmifolia*	
Lamb shank (*n* = 18)	*Anadenanthera falcate*		
Chicken breast (without skin) (*n* = 18)	Treated *Eucalyptus citriodora*		

*n* = number of samples.

**Table 2:** Operating conditions for the microwave digestion system.

Parameters	Step		
	1	2	3
Temperature (°C)	100	150	50
Ramp time (min)	1	1	1
Hold time (min)	5	10	1
Power (W)	1160	1160	0
Pressure (Bar)	30	30	25

**Table 3:** ICP-OES instrumental parameters.

Parameter	Setting
Sample Flush Time (s)	30
Pump Stabilization Time (s)	5.0
Nebulizer Gas Flow (L/min)	0.7
Auxiliary Gas Flow (L/min)	0.5
Flush Pump Rate (rpm)	50
RF Power (W)	1150
Analysis Pump Rate (rpm)	50
Coolant Gas Flow (L/min)	12
Analytes/λ (nm)	Al 167.079; As 189.042; Cd 228.802; Co 228.616; Cr 283.563; Cu 324.754; Fe 259.940; Mg 279.553; Mn 257.610; Mo 202.030; Ni 221.647; Pb 220.353; V 309.311; Zn 213.856.

**Table 4:** Addition recovery test on metals and metalloid.

Analyte	Spike Recovery (%)	
	0.5 mg/L	1.0 mg/L
Al	108	108
As	102	103
Cd	91	93
Co	99	99
Cr	107	108
Cu	104	104
Fe	106	105
Mg	101	99
Mn	100	101
Mo	111	112
Ni	101	101
Pb	90	93
V	110	110
Zn	102	99

**Table 5:** Calibration equations (y = ax + b) *, correlation coefficients (*R*^2^), limits of detection (LOD), and limits of quantification (LOQ) obtained by external calibration.

Element	Equation External Calibration y = ax + b	LOD (mg/L)	LOQ (mg/L)	*R* ^2^
Al	y = 136.61x − 1.1213	0.0045	0.0151	0.9994
As	y = 469.39x + 8.7286	0.0029	0.0097	0.9995
Cd	y = 13845x + 102.27	0.0002	0.0007	0.9998
Co	y = 5806.4x + 56.482	0.0005	0.0016	0.9998
Cr	y = 18,084x + 87.356	0.0011	0.0035	0.9998
Cu	y = 16,191x + 192.33	0.0013	0.0042	0.9998
Fe	y = 10,923x + 122.77	0.0011	0.0036	0.9998
Mg	y = 397,282x + 884.4	0.0007	0.0024	0.9995
Mn	y = 57509x + 609.65	0.0002	0.0005	0.9998
Mo	y = 3703.8x + 33.342	0.0005	0.0015	0.9997
Ni	y = 5338.5x + 64.394	0.0005	0.0016	0.9998
Pb	y = 1008.1x + 29.193	0.0040	0.0132	0.9998
V	y = 34,980x + 359.82	0.0004	0.0014	0.9998
Zn	y = 10,414x + 130.04	0.0004	0.0014	0.9998

* y = intensity; a = slop; x = concentration (mg/L); b = intercept.

**Table 6:** Trace (Al, As, Cd, Cr, Cu, Fe, Mn, Mo, Ni, V, and Zn) and major (Mg) elements in different meat raw and roasted beef, pork, lamb, and chicken using different fuels.

Element	Concentrations (Average Weight mg/kg ± SD)							
	Raw Meat	Electric Grill Burgers	*Eucalyptus citriodora* Wood	*Guazuma ulmifolia* Wood	*Anadenanthera falcata* Wood	CCA-Treated Eucalyptus	*Eucalyptus citriodora* Coal	*Guazuma ulmifolia* Coal
**Al**								
Beef topside	1.25 ± 0.23 ^a^	9.78 ± 1.69 ^d^	3.31 ± 0.57 ^a,b^	6.53 ± 1.52 ^b,c^	17.49 ± 0.23 ^f^	11.61 ± 0.60 ^e^	7.28 ± 0.66 ^c^	27.79 ± 0.71 ^g^
Pork loin	0.86 ± 0.12 ^a^	25.53 ± 0.21 ^f^	2.85 ± 0.50 ^b^	8.39 ± 0.10 ^c^	15.33 ± 0.46 ^e^	11.59 ± 0.59 ^d^	9.11 ± 0.67 ^c^	56.90 ± 0.73 ^g^
Lamb shank	1.54 ± 0.55 ^a^	16.32 ± 0.25 ^d^	2.89 ± 0.05 ^a^	15.19 ± 0.33 ^c,d^	12.12 ± 0.64 ^c^	17.18 ±1.92 ^d^	6.56 ± 0.13 ^b^	28.22 ± 0.64 ^e^
Chicken breast	1.01 ± 0.15 ^a^	37.30 ± 0.19 ^e^	1.81 ± 0.13 ^a^	12.53 ± 0.99 ^c^	23.12 ± 0.86 ^d^	12.91 ± 0.32 ^c^	6.12 ± 0.50 ^b^	46.46 ± 1.03 ^f^
**As**								
Beef topside	0.23 ± 0.10 ^a^	0.55 ± 0.12 ^a^	0.55 ± 0.45 ^a^	0.29 ± 0.03 ^a^	0.33 ± 0.05 ^a^	21.04 ± 0.14 ^b^	1.16 ± 0.50 ^a^	0.47 ± 0.24 ^a^
Pork loin	0.17 ± 0.42 ^a^	0.55 ± 0.04 ^a^	0.30 ± 0.23 ^a^	0.40 ± 0.01 ^a^	0.19 ± 0.04 ^a^	15.10 ± 0.27 ^b^	0.83 ± 0.60 ^a^	1.04 ± 0.65 ^a^
Lamb shank	ND	0.67 ± 0.07 ^a^	0.64 ± 0.18 ^a^	0.20 ± 0.20 ^a^	0.10 ± 0.02 ^a^	17.61 ± 0.50 ^b^	0.59 ± 0.25 ^a^	0.46 ± 0.09 ^a^
Chicken breast	0.19 ± 0.36 ^a^	0.57 ± 0.02 ^a^	0.55 ± 0.53 ^a^	0.37 ± 0.06 ^a^	0.37 ± 0.01 ^a^	26.25 ± 1.47 ^b^	0.59 ± 0.57 ^a^	0.93 ± 0.29 ^a^
**Cd**								
Beef topside	ND	0.09 ± 0.03 ^a^	ND	ND	ND	ND	ND	ND
Pork loin	ND	0.11 ± 0.04 ^a^	ND	ND	ND	ND	ND	ND
Lamb shank	ND	ND	ND	ND	ND	ND	ND	ND
Chicken breast	ND	0.12 ± 0.01 ^a^	ND	ND	ND	ND	ND	ND
**Cr**								
Beef topside	0.15 ± 0.24 ^a^	1.32 ± 0.09 ^b^	ND	ND	ND	1.10 ± 0.05 ^b^	0.004 ± 0.001 ^a^	0.66 ± 0.55 ^a,b^
Pork loin	ND	0.94 ± 0.02 ^b^	ND	0.14 ± 0.02 ^a^	0.16 ± 0.00 ^a^	0.79 ± 0.16 ^b^	0.08 ± 0.012 ^b^	0.13 ± 0.014 ^a^
Lamb shank	ND	0.25 ± 0.06 ^b^	ND	0.09 ± 0.00 ^a^	0.03 ± 0.04 ^a^	0.90 ± 0.04 ^c^	0.03 ± 0.05 ^a^	ND
Chicken breast	ND	0.55 ± 0.01 ^a,b^	ND	0.10 ± 0.02 ^a^	0.10 ± 0.08 ^a,b^	0.89 ± 0.37 ^b^	ND	0.14 ± 0.11 ^a^
**Cu**								
Beef topside	0.29 ± 0.03 ^a^	0.94 ± 0.00 ^b^	2.31 ± 0.15 ^d^	1.79 ± 0.04 ^c^	1.66 ± 0.02 ^c^	2.10 ± 0.01 ^c,d^	1.28 ± 0.14 ^c^	1.22 ± 0.01 ^b,c^
Pork loin	ND	0.53 ± 0.01 ^b^	0.16 ± 0.00 ^a^	0.17 ± 0.02 ^a^	0.13 ± 0.01 ^a^	0.71 ± 0.16 ^b^	0.17 ± 0.06 ^a^	0.33 ± 0.12 ^b^
Lamb shank	0.84 ± 0.01 ^a^	1.95 ± 0.12 ^c^	1.04 ± 0.09 ^a,b^	1.29 ± 0.07 ^b^	0.83 ± 0.02 ^a^	2.80 ± 0.02 ^d^	1.97 ± 0.24 ^c^	2.10 ± 0.09 ^c^
Chicken breast	ND	0.23 ± 0.01 ^a^	ND	0.19 ± 0.00 ^a^	0.09 ± 0.02 ^a^	0.63 ± 0.22 ^b^	ND	0.23 ± 0.04 ^a^
**Fe**								
Beef topside	37.52 ± 1.58 ^a^	48.59 ± 1.06 ^a,b^	37.87 ± 2.01 ^a^	33.73 ± 0.05 ^a^	36.54 ± 0.47 ^a^	69.34 ± 0.18 ^b,c^	82.03 ± 16.08 ^c^	87.08 ± 0.51 ^c^
Pork loin	5.18 ± 0.26 ^a^	8.52 ± 0.52 ^a^	8.13 ± 0.24 ^a^	11.68 ± 0.81 ^a^	12.06 ± 0.50 ^a^	9.18 ± 0.40 ^b^	8.95 ± 1.27 ^b^	11.58 ± 1.19 ^b^
Lamb shank	18.76 ± 0.19 ^a^	26.97 ± 3.22 ^a,b^	50.30 ± 4.69 ^c^	71.71 ± 5.36 ^d^	58.84 ± 0.48 ^c^	38.54 ± 2.30 ^b^	34.43 ± 2.24 ^b^	35.79 ± 0.38 ^b^
Chicken breast	4.58 ± 0.76 ^a^	5.33 ± 0.12 ^a^	7.82 ± 0.11 ^b^	13.02 ± 0.34 ^c,d^	15.65 ± 0.54 ^d^	10.74 ± 0.28 ^b,c^	6.92 ± 0.34 ^a,b^	19.35 ± 2.15 ^e^
**Mg**								
Beef topside	218.47 ± 3.95 ^a^	267.62 ± 2.95 ^b^	346.86 ± 19.23 ^c^	307.96 ± 2.89 ^b,c^	321.02 ± 0.90 ^c^	346.76 ± 7.66 ^c^	370.23 ± 17.04 ^c^	355.36 ± 13.18 ^c^
Pork loin	225.55 ± 8.08 ^a^	293.32 ± 1.58 ^b^	322.29 ± 6.61 ^b,c^	354.44 ± 3.91 ^c^	335.53 ± 1.90 ^b,c^	331.11 ± 10.49 ^b,c^	366.53 ± 24.81 ^c^	205.79 ± 29.193 ^a^
Lamb shank	206.77 ± 3.99 ^a^	252.79 ± 4.58 ^b^	305.53 ± 8.56 ^c^	359.58 ± 13.99 ^d^	287.79 ± 0.55 ^b,c^	342.67 ± 11.25 ^c,d^	317.62 ± 18.47 ^c,d^	332.96 ± 13.80 ^c,d^
Chicken breast	291.95 ± 8.87 ^a^	289.48 ± 0.73 ^a^	345.90 ± 1.12 ^b^	418.78 ± 12.02 ^c^	407.88 ± 3.07 ^c^	414.27 ± 1.44 ^c^	333.30 ± 2.32 ^b^	412.22 ± 0.04 ^c^
**Mn**								
Beef topside	ND	0.14 ± 0.00 ^a,b^	0.96 ± 0.07 ^c^	0.004 ± 0.02 ^a^	0.25 ± 0.02 ^b^	0.14 ± 0.01 ^a,b^	0.86 ± 0.08 ^c^	0.22 ± 0.04 ^b^
Pork loin	ND	0.10 ± 0.00 ^a,b^	0.57 ± 0.01 ^b,c^	0.02 ± 0.03 ^a^	ND	ND	1.00 ± 0.36 ^c^	0.11 ± 0.02 ^a,b^
Lamb shank	ND	0.13 ± 0.01 ^a^	0.55 ± 0.06 ^b^	0.07 ± 0.05 ^a^	0.02 ± 0.00 ^a^	0.12 ± 0.02 ^a^	0.53 ± 0.11 ^b^	0.18 ± 0.04 ^a^
Chicken breast	ND	0.11 ± 0.00 ^a^	0.55 ± 0.01 ^e^	0.12 ± 0.04 ^b^	0.27 ± 0.01 ^c^	0.10 ± 0.02 ^b^	0.27 ± 0.01 ^c^	0.45 ± 0.03 ^d^
**Mo**								
Beef topside	ND	ND	0.73 ± 0.01 ^c^	0.58 ± 0.01 ^b^	0.55 ± 0.00 ^a^	ND	ND	ND
Pork loin	ND	ND	ND	ND	ND	ND	ND	ND
Lamb shank	0.45 ± 0.05 ^a^	1.00 ± 0.04 ^c^	ND	ND	ND	0.67 ± 0.04 ^b^	0.87 ± 0.11 ^b,c^	0.92 ± 0.07 ^c^
Chicken breast	ND	0.01 ± 0.00 ^a^	ND	ND	ND	ND	ND	ND
**Ni**								
Beef topside	ND	0.49 ± 0.02 ^b^	ND	ND	ND	ND	ND	ND
Pork loin	ND	1.12 ± 0.33 ^b^	ND	ND	ND	ND	ND	ND
Lamb shank	ND	0.72 ± 0.19 ^b^	ND	ND	ND	ND	ND	ND
Chicken breast	ND	0.61 ± 0.05 ^b^	ND	ND	ND	ND	ND	ND
**V**								
Beef topside	0.42 ± 0.14 ^a^	0.84 ± 0.01 ^b,c^	0.97 ± 0.03 ^b,c^	0.73 ± 0.0 ^b^	0.80 ± 0.02 ^b^^,c^	0.98 ± 0.02 ^b,c^	1.02 ± 0.06 ^c^	1.08 ± 0.09 ^c^
Pork loin	0.39 ± 0.02 ^a^	0.96 ± 0.00 ^b^	0.81 ± 0.02 ^b^	0.97 ± 0.10 ^b^	0.83 ± 0.03 ^b^	0.88 ± 0.10 ^b^	1.04 ± 0.13 ^b,c^	1.34 ± 0.15 ^c^
Lamb shank	0.55 ± 0.36 ^a^	0.78 ± 0.02 ^a^	0.70 ± 0.07 ^a^	0.97 ± 0.09 ^a^	0.64 ± 0.02 ^a^	0.94 ± 0.06 ^a^	0.87 ± 0.07 ^a^	0.92 ± 0.03 ^a^
Chicken breast	6.66 ± 0.80 ^a^	9.30 ± 0.02 ^b,c^	8.73 ± 0.29 ^a,b^	13.82 ± 1.06 ^d^	12.27 ± 0.07 ^c,d^	11.33 ± 0.20 ^c^	9.15 ± 0.59 ^b^	16.29 ± 0.40 ^e^
**Zn**								
Beef topside	48.13 ± 0.56 ^a^	70.56 ± 1.41 ^b^	46.73 ± 2.39 ^a^	48.33 ± 0.23 ^a^	47.98 ± 0.57 ^a^	87.65 ± 0.58 ^c^	92.25 ± 10.29 ^c^	115.24 ±1.26 ^d^
Pork loin	10.97 ± 0.26 ^a^	18.07 ± 0.27 ^b^	15.93 ± 0.76 ^b^	17.61 ± 1.24 ^b^	15.03 ± 0.42 ^b^	15.67 ± 0.50 ^b^	17.78 ± 1.90 ^b^	21.16 ± 1.22 ^c^
Lamb shank	38.70 ± 0.11 ^a^	62.40 ± 0.58 ^c^	65.56 ± 2.82 ^c^^,d^	81.14 ± 3.18 ^e^	71.74 ± 1.02 ^d^	47.62 ± 1.99 ^b^	45.80 ± 3.67 ^a,b^	76.97 ± 1.53 ^d,e^
Chicken breast	6.66 ± 0.80 ^a^	8.05 ± 0.17 ^a,b^	8.73 ± 0.29 ^a,b^	13.82 ± 1.06 ^c^	12.27 ± 0.07 ^c^	11.33 ± 0.20 ^b,c^	9.15 ± 0.59 ^b^	16.29 ± 0.40 ^d^

Notes: Different letters in the same line represent statistic differences amongst groups (*p* < 0.05) by one-way ANOVA with post hoc Tukey’s test. ND: not detectable; values ND assumed to be zero. CCA = Chromed Copper Arsenate.

**Table 7:** Estimated daily intakes (*EDI*, µg/kg bw/day) and hazard quotient (*HQ*) of elements through roasted beef topside and roasted pork loin consumption in Brazil.

Sample		Al	As	Cd	Cr	Cu	Fe	Mg	Mn	Mo	Ni	V	Zn
roasted beef topside with electric grill	*EDI*	8.8299	0.4966	0.0813	1.1918	0.8487	43.8698	241.6226	0.1264	-	0.4424	0.7584	63.7056
	*HQ*	22.0749	1.6552	0.0813	0.3973	-	0.0627	0.2197	0.0009	-	0.0221	0.0843	0.2124
roasted beef topside with wood fire from *Eucalyptus citriodora*	*EDI*	2.9885	0.4966	-	-	2.0856	34.1912	313.1650	0.8667	0.6591	-	0.8758	42.1905
	*HQ*	7.4711	1.6552	-	-	-	0.0488	0.0285	0.0062	0.1318	-	0.0973	0.1406
roasted beef topside with wood fire from *Guazuma ulmifolia*	*EDI*	5.8957	0.2618	-	-	1.6161	30.4534	278.0439	0.0036	0.5237	-	0.6591	43.6351
	*HQ*	14.7391	0.8728	-	-	-	0.0435	0.0253	0.00003	0.1047	-	0.0732	0.1455
roasted beef topside with wood fire from *Anadenanthera falcate*	*EDI*	15.7910	0.2979	-	-	1.4987	32.9904	289.8352	0.2257	0.4966	-	0.7223	43.3191
	*HQ*	39.4774	0.9931	-	-	-	0.0471	0.0263	0.0016	0.0993	-	0.0803	0.1444
roasted beef topside with wood fire from CCA-treated *Eucalyptus*	*EDI*	10.4822	18.9961	-	0.9931	1.8960	62.6041	313.0747	0.1264	-	-	0.8848	79.1354
	*HQ*	26.2054	63.3204	-	0.3310	-	0.0894	0.0285	0.0009	-	-	0.0983	0.2638
roasted beef topside with coal fire from *Eucalyptus citriodora*	*EDI*	6.5728	1.0473	-	0.0036	1.1557	74.0614	334.2648	0.7765	-	-	0.9209	83.2886
	*HQ*	16.4320	3.4910	-	0.0012	-	0.1058	0.0304	0.0055	-	-	0.1023	0.2776
roasted beef topside with coal fire from *Guazuma ulmifolia*	*EDI*	25.2078	0.4243	-	0.5959	1.1015	78.6208	320.8393	0.1986	-	-	0.9751	104.0453
	*HQ*	63.0194	1.4145	-	0.1986	-	0.1123	0.0292	0.0014	-	-	0.1083	0.3468
roasted pork loin with electric grill	*EDI*	3.1001	0.0668	0.0134	0.1141	0.0644	1.0346	35.6174	0.0121	-	0.1360	0.1166	2.1942
	*HQ*	7.7502	0.2226	0.0134	0.0380	-	0.0015	0.0324	0.0001	-	0.0068	0.0130	0.0073
roasted pork loin with wood fire from *Eucalyptus citriodora*	*EDI*	0.3461	0.0364	-	-	0.0194	0.9872	39.1352	0.0692	-	-	0.0984	1.9344
	*HQ*	0.8652	0.1214	-	-	-	0.0014	0.0036	0.0005	-	-	0.0109	0.0064
roasted pork loin with wood fire from *Guazuma ulmifolia*	*EDI*	1.0188	0.0486	-	0.0170	0.0206	1.4183	43.0391	0.0024	-	-	0.1178	2.1384
	*HQ*	2.5470	0.1619	-	0.0057	-	0.0020	0.0039	0.00002	-	-	0.0131	0.0071
roasted pork loin with wood fire from *Anadenanthera falcate*	*EDI*	1.8615	0.0231	-	0.0194	0.0158	1.4644	40.7429	-	-	-	0.1008	1.8251
	*HQ*	4.6538	0.0769	-	0.0065	-	0.0021	0.0037	-	-	-	0.0112	0.0061
roasted pork loin with wood fire from CCA-treated *Eucalyptus*	*EDI*	1.4074	1.8336	-	0.0959	0.0862	1.1147	40.2062	-	-	-	0.1069	1.9028
	*HQ*	3.5184	6.1119	-	0.0320	-	0.0016	0.0037	-	-	-	0.0119	0.0063
roasted pork loin with coal fire from *Eucalyptus citriodora*	*EDI*	1.1062	0.1008	-	0.0097	0.0206	1.0868	44.5072	0.1214	-	-	0.1263	2.1590
	*HQ*	2.7655	0.3360	-	0.0032	-	0.0016	0.0040	0.0009	-	-	0.0140	0.0072
roasted pork loin with coal fire from *Guazuma ulmifolia*	*EDI*	6.9093	0.1263	-	0.0158	0.0401	1.4061	24.9888	0.0134	-	-	0.1627	2.5694
	*HQ*	17.2732	0.4210	-	0.0053	-	0.0020	0.0023	0.0001	-	-	0.0181	0.0086

**Table 8:** Estimated daily intakes (*EDI*, µg/kg bw/day) and hazard quotient (*HQ*) of elements through roasted chicken breast and roasted lamb shank in Brazil.

Sample		Al	As	Cd	Cr	Cu	Fe	Mg	Mn	Mo	Ni	V	Zn
roasted chicken breast with electric grill	*EDI*	19.4493	0.2972	0.0626	0.2868	0.1199	2.7792	150.9431	0.0574	0.0052	0.3181	4.8493	4.1975
	*HQ*	48.6232	0.9907	0.0626	0.0956	-	0.0040	0.1372	0.0004	0.0010	0.0159	0.5388	0.0140
roasted chicken breast with wood fire from *Eucalyptus citriodora*	*EDI*	0.9438	0.2868	-	-	-	4.0776	180.3621	0.2868	-	-	0.4745	4.5521
	*HQ*	2.3595	0.9560	-	-	-	0.0058	0.0164	0.0020	-	-	0.0527	0.0152
roasted chicken breast with wood fire from *Guazuma ulmifolia*	*EDI*	6.5335	0.1929	-	0.0521	0.0991	6.7890	218.3639	0.0626	-	-	0.7821	7.2061
	*HQ*	16.3338	0.6431	-	0.0174	-	0.0097	0.0199	0.0004	-	-	0.0869	0.0240
roasted chicken breast with wood fire from *Anadenanthera falcate*	*EDI*	12.0554	0.1929	-	0.0521	0.0469	8.1604	212.6803	0.1408	-	-	0.6883	6.3979
	*HQ*	30.1386	0.6431	-	0.0174	-	0.0117	0.0193	0.0010	-	-	0.0765	0.0213
roasted chicken breast with wood fire from CCA-treated *Eucalyptus*	*EDI*	6.7316	13.6875	-	0.4641	0.3285	5.6001	216.0122	0.0521	-	-	0.7196	5.9078
	*HQ*	16.8291	45.6250	-	0.1547	-	0.0080	0.0196	0.0004	-	-	0.0800	0.0197
roasted chicken breast with coal fire from *Eucalyptus citriodora*	*EDI*	3.1911	0.3076	-	-	-	3.6083	173.7921	0.1408	-	-	0.4536	4.7711
	*HQ*	7.9779	1.0255	-	-	-	0.0052	0.0158	0.0010	-	-	0.0504	0.0159
roasted chicken breast with coal fire from *Guazuma ulmifolia*	*EDI*	24.2256	0.4849	-	0.0730	0.1199	10.0896	214.9433	0.2346	-	-	0.9021	8.4941
	*HQ*	60.5639	1.6164	-	0.0243	-	0.0144	0.0195	0.0017	-	-	0.1002	0.0283
roasted lamb shank with electric grill	*EDI*	0.1865	0.0077	-	0.1768	0.0029	0.0223	0.3082	2.8890	0.0015	0.0114	0.0089	0.7131
	*HQ*	0.4663	0.0255	-	0.4420	-	-	0.0004	0.0026	0.0000	0.0023	0.0010	0.0024
roasted lamb shank with wood fire from *Eucalyptus citriodora*	*EDI*	0.0330	0.0073	-	-	0.0119	0.5749	3.4918	0.0063	-	-	0.0080	0.7493
	*HQ*	0.0826	0.0244	-	-	-	0.0008	0.0003	0.00004	-	-	0.0009	0.0025
roasted lamb shank with wood fire from *Guazuma ulmifolia*	*EDI*	0.1736	0.0023	-	0.0010	0.0147	0.8195	4.1095	0.0008	-	-	0.0111	0.9273
	*HQ*	0.4340	0.0076	-	0.0003	-	0.0012	0.0004	0.00001	-	-	0.0012	0.0031
roasted lamb shank with wood fire from *Anadenanthera falcate*	*EDI*	0.1385	0.0011	-	0.0003	0.0095	0.6725	3.2890	0.0002	-	-	0.0073	0.8199
	*HQ*	0.3462	0.0038	-	0.0001	-	0.0010	0.0003	0.000002	-	-	0.0008	0.0027
roasted lamb shank with wood fire from CCA-treated *Eucalyptus*	*EDI*	0.1963	0.2013	-	0.0103	0.0320	0.4405	3.9162	0.0014	0.0077	-	0.0107	0.5442
	*HQ*	0.4909	0.6709	-	0.0034	-	0.0006	0.0004	0.00001	0.0015	-	0.0012	0.0018
roasted lamb shank coal fire from *Eucalyptus citriodora*	*EDI*	0.0750	0.0067	-	0.0003	0.0225	0.3935	3.6299	0.0061	0.0099	-	0.0099	0.5234
	*HQ*	0.1874	0.0225	-	0.0001	-	0.0006	0.0003	0.00004	0.0020	-	0.0011	0.0017
roasted lamb shank coal fire from *Guazuma ulmifolia*	*EDI*	0.3225	0.0053	-	-	0.0240	0.4090	3.8053	0.0021	0.010	-	0.0105	0.8797
	*HQ*	0.8063	0.0175	-	-	-	0.0006	0.0003	0.00001	0.0021	-	0.0012	0.0029

## Supplementary Materials

The following are available online at https://www.mdpi.com/1660-4601/17/18/6737/s1. Figure S1: Comparison of mineral concentration in raw and roasted beef, pork, lamb and chicken using different fuels. (**a**) Aluminum, (**b**) Arsenium, (**c**) Chromium, (**d**) Copper, (**e**) Iron, (**f**) Magnesium, (**g**) Manganese, (**h**) Molybdenum, (**i**) Vanadium, (**j**) Zinc, (**k**) Cadmium, (**l**) Nickel.
