# Supplementary material for: Human Health Risk Assessment through Roasted Meats Consumption
Source: Int J Environ Res Public Health. 2020 Sep 16;17(18):6737. doi: 10.3390/ijerph17186737 (PMC7558450; doi:10.3390/ijerph17186737)
Supplement: Supplementary file 1 [file ijerph-17-06737-s001.pdf]

International Journal of *Environmental Research and Public Health*

MDPI

Supplementary Materials: Human Health Risk Assessment through Roasted Meats Consumption

(**a**)

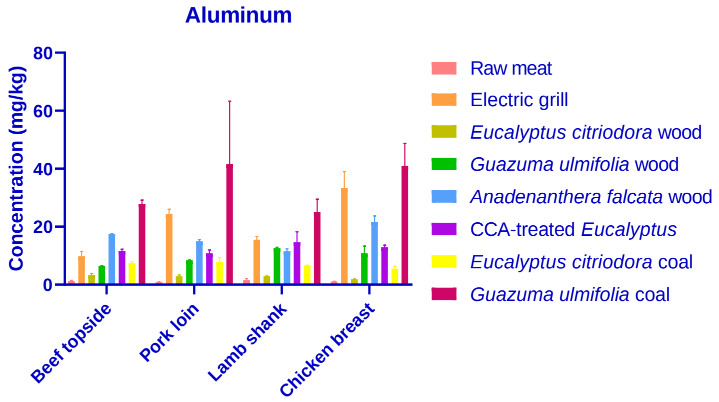

(**b**)

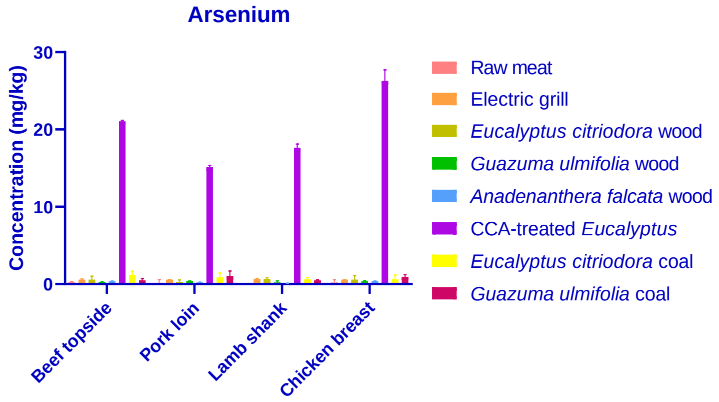

(**c**)

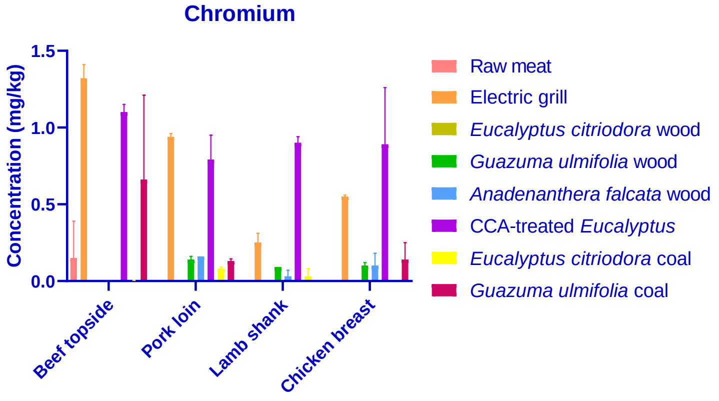

(**d**)

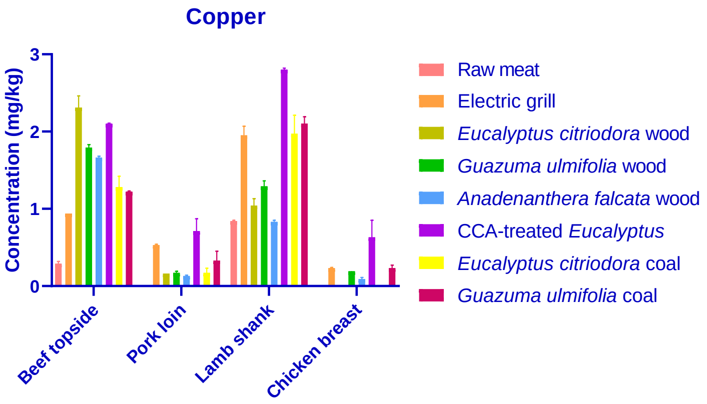
Copper
Concentration (mg/kg)
0
1
2
3
Beef topside
Pork loin
Lamb shank
Chicken breast
Raw meat
Electric grill
Eucalyptus citriodora wood
Guazuma ulmifolia wood
Anadenanthera falcata wood
CCA-treated Eucalyptus
Eucalyptus citriodora coal
Guazuma ulmifolia coal

(**e**)

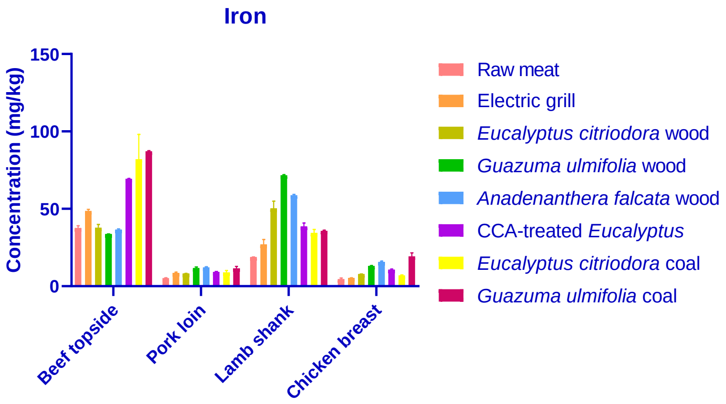
Iron
Concentration (mg/kg)
0
50
100
150
Beef topside
Pork loin
Lamb shank
Chicken breast
Raw meat
Electric grill
Eucalyptus citriodora wood
Guazuma ulmifolia wood
Anadenanthera falcata wood
CCA-treated Eucalyptus
Eucalyptus citriodora coal
Guazuma ulmifolia coal

(**f**)

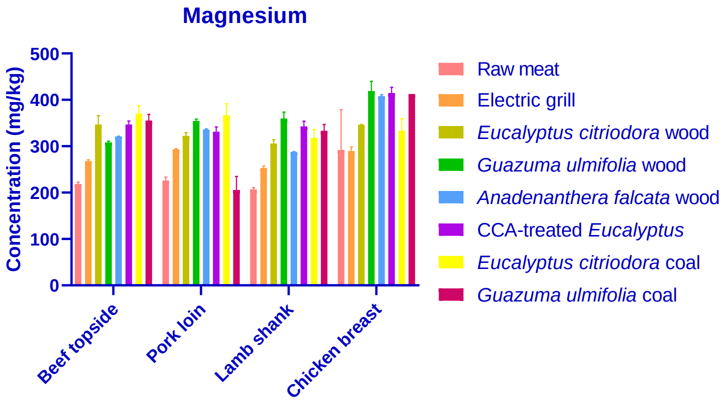
Magnesium
Concentration (mg/kg)
0
100
200
300
400
500
Beef topside
Pork loin
Lamb shank
Chicken breast
Raw meat
Electric grill
Eucalyptus citriodora wood
Guazuma ulmifolia wood
Anadenanthera falcata wood
CCA-treated Eucalyptus
Eucalyptus citriodora coal
Guazuma ulmifolia coal

(**g**)

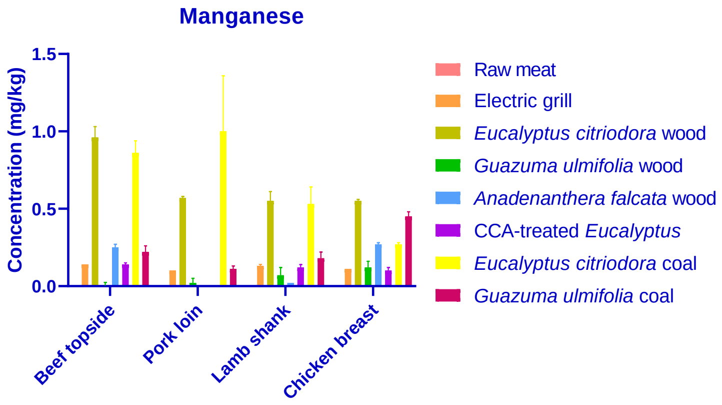
Manganese
Concentration (mg/kg)
0.0
0.5
1.0
1.5
Beef topside
Pork loin
Lamb shank
Chicken breast
Raw meat
Electric grill
Eucalyptus citriodora wood
Guazuma ulmifolia wood
Anadenanthera falcata wood
CCA-treated Eucalyptus
Eucalyptus citriodora coal
Guazuma ulmifolia coal

(**h**)

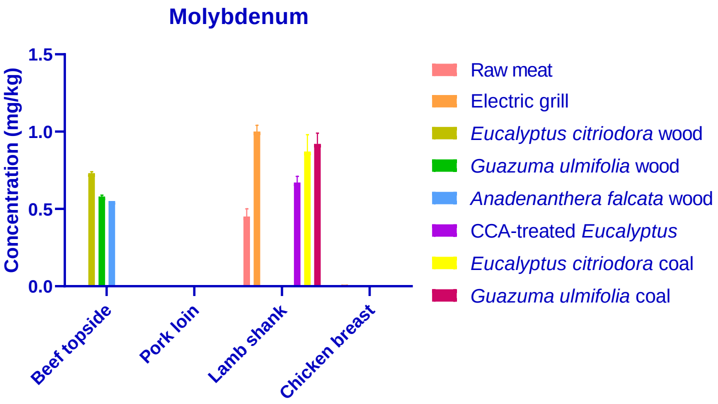
Molybdenum
Concentration (mg/kg)
1.5
1.0
0.5
0.0
Beef topside
Pork loin
Lamb shank
Chicken breast
Raw meat
Electric grill
Eucalyptus citriodora wood
Guazuma ulmifolia wood
Anadenanthera falcata wood
CCA-treated *Eucalyptus*
Eucalyptus citriodora coal
Guazuma ulmifolia coal

(**i**)

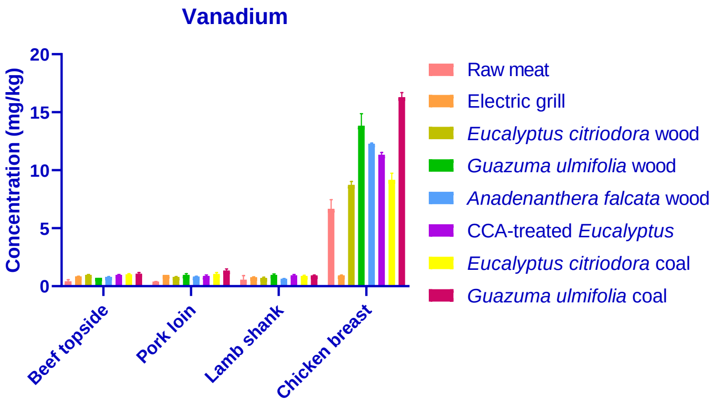
Vanadium
Concentration (mg/kg)
20
15
10
5
0
Beef topside
Pork loin
Lamb shank
Chicken breast
Raw meat
Electric grill
Eucalyptus citriodora wood
Guazuma ulmifolia wood
Anadenanthera falcata wood
CCA-treated *Eucalyptus*
Eucalyptus citriodora coal
Guazuma ulmifolia coal

(**j**)

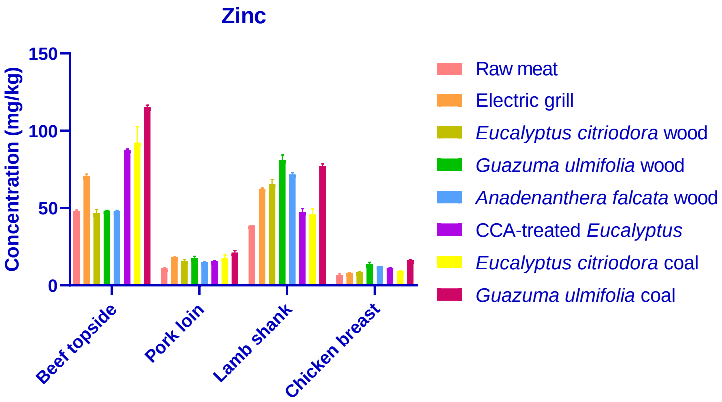
Zinc
Concentration (mg/kg)
150
100
50
0
Beef topside
Pork loin
Lamb shank
Chicken breast
Raw meat
Electric grill
Eucalyptus citriodora wood
Guazuma ulmifolia wood
Anadenanthera falcata wood
CCA-treated *Eucalyptus*
Eucalyptus citriodora coal
Guazuma ulmifolia coal

(**k**)

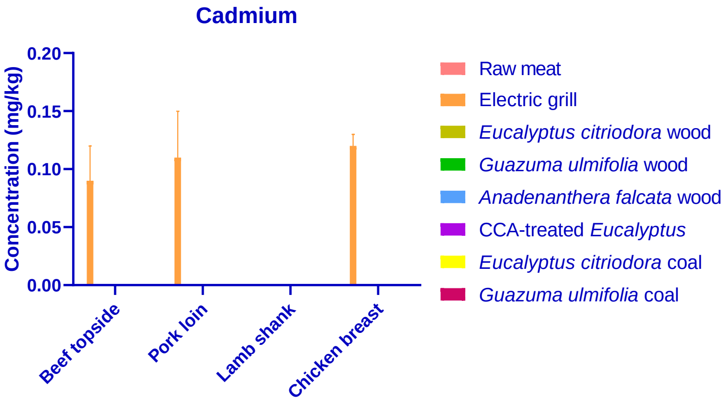
Cadmium
Concentration (mg/kg)
0.20
0.15
0.10
0.05
0.00
Beef topside
Pork loin
Lamb shank
Chicken breast
Raw meat
Electric grill
Eucalyptus citriodora wood
Guazuma ulmifolia wood
Anadenanthera falcata wood
CCA-treated *Eucalyptus*
Eucalyptus citriodora coal
Guazuma ulmifolia coal

(**l**)

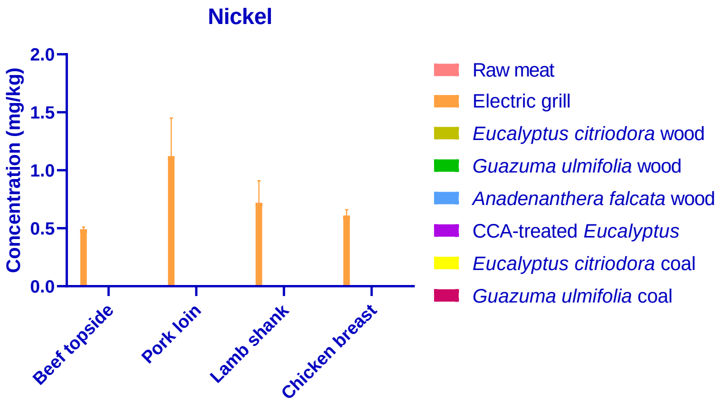

Figure S1. Comparison of mineral concentration in raw and roasted beef, pork, lamb and chicken using different fuels. (**a**) Aluminum, (**b**) Arsenium, (**c**) Chromium, (**d**) Copper, (**e**) Iron, (**f**) Magnesium, (**g**) Manganese, (**h**) Molybdenum, (**i**) Vanadium, (**j**) Zinc, (**k**) Cadmium, (**l**) Nickel.

© 2020 by the authors. Submitted for possible open access publication under the terms and conditions of the Creative Commons Attribution (CC BY) license (http://creativecommons.org/licenses/by/4.0/).
